# Supplementary material for: Minimal Inhibitory Concentration (MIC)-Phenomena in Candida albicans and Their Impact on the Diagnosis of Antifungal Resistance
Source: J Fungi (Basel). 2019 Sep 4;5(3):83. doi: 10.3390/jof5030083 (PMC6787722; doi:10.3390/jof5030083)
Supplement: Supplementary file 1 [file jof-05-00083-s001.pdf]

SUPPLEMENTS

Table S1. Overview on the clinical isolates of *C. albicans* showing MIC-phenomena determined by EUCAST assay.

Strain ID	Species	Antifungal drug	MIC 24 h µg/mL	MIC phenomena (µg/mL)
SCH 24	*C. albicans*	VOR	0.06	trailing[a] > 8
		FLU	0.5	trailing[a] > 32
		POS	0.03	trailing[a] > 8
		ITR	0.125	trailing[a] > 16
		CAS	0.12	
		ANI	0.03	
		AMB	0.25	
SCH 36	*C. albicans*	VOR	0.01	trailing[a] > 8
		FLU	1	trailing[a] > 32
		POS	0.01	trailing[a] > 8
		ITR	0.125	trailing[a] > 16
		CAS	0.06	paradoxical growth[b] at 4 and 8
		ANI	0.03	
		AMB	0.25	
SCH 40	*C. albicans*	VOR	0.01	trailing[a] > 8
		FLU	1	trailing[a] > 32
		POS	0.01	trailing[a] > 8
		ITR	0.125	trailing[a] > 16
		CAS	0.03	paradoxical growth[b] at 4 and 8
		ANI	0.03	paradoxical growth[b] at 4
		AMB	0.25	
SCH12		VOR	0.06	
		POS	0.03	trailing[a] > 8
		CAS	0.12	
		ANI	0.03	
SCH16		VOR	0.01	
		POS	0.01	
		CAS	0.06	paradoxical growth[b] at 4 and 8
		ANI	0.03	
SCH14		VOR	0.01	trailing[a] > 8
		POS	0.01	
		CAS	0.03	paradoxical growth[b] at 4 and 8
		ANI	0.03	
SCH22		VOR	0.06	
		POS	0.03	trailing[a] > 8
		CAS	0.12	

	ANI	0.03	
	VOR	0.01	
	POS	0.01	
SCH26	CAS	0.06	
	ANI	0.03	paradoxical growth[b] at 4

VOR, voriconazole; FLU, fluconazole; ITR, itraconazole, POS, posaconazole; CAS, caspofungin; ANI, anidulafungin; MICA, micafungin; AMB amphotericin B; [a]Trailing is defined as a reduced turbidity compared to the positive growth control, fully inhibited growth is not reacted and therefore well fails to become optically clear for MIC reading (as suggested by the guideline).; [b]Paradoxical phenomenon (PXE) was defined as reoccurring growth at higher concentrations than the determined MIC.

Figure S1

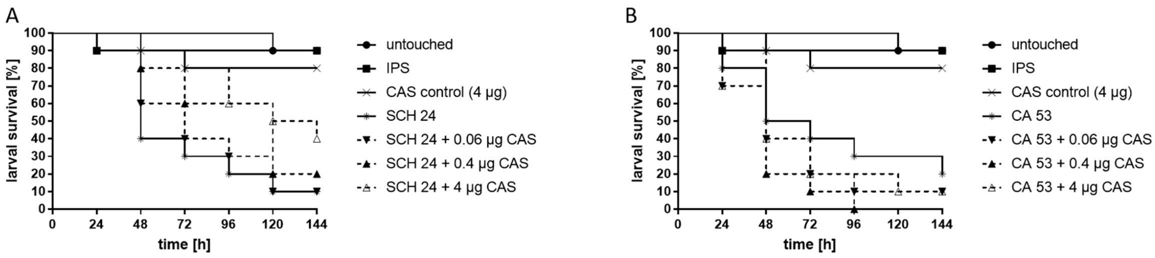

Figure S1. *In vivo* efficacy of caspofungin (CAS) in larvae infected with CAS susceptible (A) and CAS resistant (B) strains (no MIC phenomenon). Caspofungin treatment significantly increased ($p \leq 0.05$) survival of *G. mellonella* larvae infected with *C. albicans* SCH 24 (= susceptible strain) and exhibited no curative effect on larvae infected with CR 53 (= resistant strain). Larvae were infected with each 10^6 cells and received antifungal treatment 2h post infection. Insect physiological saline (IPS) was used as an injection control and larvae receiving no treatment served as control.

Figure S2

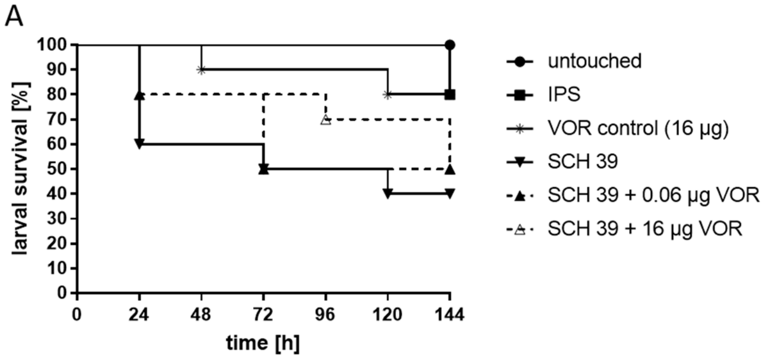

Figure S2. Voriconazole (VOR) treatment increased survival of *G. mellonella* larvae although lacking statistical significance ($p = 0.48$) infected with *Candida albicans* strain SCH 39 (VOR MIC 0.0078 µg/mL). Larvae were infected with each 10^6 cells and received antifungal treatment 2h post infection. Insect physiological saline (IPS) was used as an injection control and larvae receiving no treatment served as control.
